# Collaborative care vs consultation liaison for depression and anxiety disorders in general practice: study protocol for two randomized controlled trials (the Danish Collabri Flex trials)

**DOI:** 10.1186/s13063-019-3657-0

**Published:** 2019-10-25

**Authors:** Nadja Kehler Curth, Ursula Brinck-Claussen, Kirstine Bro Jørgensen, Susanne Rosendal, Carsten Hjorthøj, Merete Nordentoft, Lene Falgaard Eplov

**Affiliations:** 1 Research Unit, Mental Health Center Copenhagen, Mental Health Services, Capital Region of Denmark, Kildegårdsvej 28, 15A, 4th floor, DK-2900 Hellerup, Denmark; 2 Mental Health Center Copenhagen, Mental Health Services, Capital Region of Denmark, Nordre Fasanvej 59, Vej 5, 12, 2nd floor, DK-2000 Frederiksberg, Denmark

**Keywords:** Collaborative care, Anxiety, Depression, Randomized controlled trial, General practice, Cognitive behavioral therapy

## Abstract

**Background:**

Models of collaborative care and consultation liaison propose organizational changes to improve the quality of care for people with common mental disorders, such as anxiety and depression. Some literature suggests only short-term positive effects of consultation liaison on patient-related outcomes, whereas collaborative care demonstrates both short-term and long-term positive effects. To our knowledge, only one randomized trial has compared the effects of these models. Collaborative care was superior to consultation liaison in reducing symptoms of depression for up to 3 months, but the authors found no difference at 9-months' follow-up. The Collabri Flex Trial for Depression and the Collabri Flex Trial for Anxiety aim to compare the effects of collaborative care with those of a form of consultation liaison that contains potential contaminating elements from collaborative care. The trials build on knowledge from the previous cluster-randomized Collabri trials.

**Methods:**

Two randomized, investigator-initiated, parallel-group, superiority trials have been established: one investigating the effects of collaborative care vs consultation liaison for depression and one investigating the effects of collaborative care vs consultation liaison for generalized anxiety, panic disorder and social anxiety disorder at 6-months' follow-up. Participants are recruited from general practices in the Capital Region of Denmark: 240 in the depression trial and 284 in the anxiety trial. The primary outcome is self-reported depression symptoms (Beck Depression Inventory (BDI-II)) in the depression trial and self-reported anxiety symptoms (Beck Anxiety Inventory (BAI)) in the anxiety trial. In both trials, the self-reported secondary outcomes are general psychological problems and symptoms (Symptom Checklist 90-Revised), functional impairment (Sheehan Disability Scale) and general well-being (World Health Organization-Five Well-Being Index). In the depression trial, BAI is an additional secondary outcome, and BDI-II is an additional secondary outcome in the anxiety trial. Explorative outcomes will also be collected.

**Discussion:**

The results will supplement those of the cluster-randomized Collabri trials and provide pivotal information about the effects of collaborative care in Denmark.

**Trial registration:**

ClinicalTrials.gov, NCT03113175 and NCT03113201. Registered on 13 April 2017.

**Electronic supplementary material:**

The online version of this article (10.1186/s13063-019-3657-0) contains supplementary material, which is available to authorized users.

## Background

Common mental disorders, such as anxiety and depression, are prevalent in the general population [1–4], contribute to high levels of morbidity and have great impact on the economy [5, 6]. According to the World Health Organization, management of these conditions should be integrated into primary care [7]. Nevertheless, it is recognized that this group of patients is both underdiagnosed and undertreated in primary care [1, 8–11]. Lack of coordination between sectors and limited availability of evidence-based treatment, such as psychotherapy, are some of the explanations for these deficiencies in Denmark [12].

To improve the quality of care for people with depression in primary care, early research focused on enhancing the primary-care providers’ knowledge and skills. Interventions such as short-term courses and passive dissemination of guidelines were generally unsuccessful in showing effects on patient outcomes [13, 14]. Later, consultation liaison interventions focused on specialist support and assistance. There is no consensus about the exact definition or content of mental health consultation liaison in the literature and the limited evidence seems inconsistent [15–17]. However, consultation liaison is broadly characterized by a mental-health worker providing specialist consultative support to a primary-care provider who has a central role in delivering mental health care. The extent of contact between the mental-health worker and the patient seems to vary according to different models of consultation liaison [17]. Using broad inclusion criteria, a Cochrane review found no statistically significant difference in symptoms from 3 to 12 months' follow-up between consultation liaison and standard care groups [17]. However, a positive effect of consultation liaison was found on mental health for up to 3 months and on treatment satisfaction and adherence for up to 12 months for different mental disorders, particularly depression [17]. Another systematic review and meta-analysis found no statistically significant improvements regarding antidepressant use or outcomes for depression in the short or long term for patients with depression [16]. The authors included only consultation liaison interventions characterized by no contact between the mental-health worker and the patient after initial assessment, and therefore used a narrower definition than did the Cochrane review.

In 2006, a set of criteria was suggested for successful system-level approaches for management of depression in primary care. These criteria are commonly referred to as criteria for collaborative care. They cover a multi-professional approach to patient care with enhanced communication between professionals, where the treatment is based on a structured management plan that includes close, scheduled follow-up [18]. Using these criteria, a Cochrane review from 2012 found that collaborative care was associated with significant improvement for up to 2 years in depression and anxiety outcomes compared with treatment as usual [19]. The impact of collaborative care has been extensively documented for depression, especially in the United States [19–21]; for anxiety disorders, collaborative care has not been as widely studied [19, 22]. Additionally, the available research differs greatly in terms of context, patient characteristics and intervention activities within the framework of collaborative care. Such differences potentially have an impact on the generalizability of results to other settings, such as Denmark [23].

In 2013, a National Health Technology Assessment (HTA) was initiated to evaluate the effect, patient satisfaction, and economic and organizational consequences of collaborative care in Denmark. As part of the HTA, a Danish collaborative care model for anxiety and depression was developed. Between 2014 and early 2017 the model was tested in the Capital Region of Denmark in four cluster-randomized superiority trials (the Collabri trials) comparing collaborative care with treatment as usual for depression, generalized anxiety disorder, social anxiety disorder and panic disorder [24, 25]. As collaborative care involves activities on the organizational level, such as ongoing supervision and support of the general practitioner (GP) by mental-health specialists, cluster randomization was chosen because of the considerable risk of control group contamination if randomization was performed on an individual level [26]. This would be likely to occur because it would be difficult for GPs to abstain from getting supervision from the mental-health specialists on patients in the control group. Despite extensive efforts, too few participants were included in the trials, especially in the control groups, which resulted in inadequate sample sizes with unequal distribution between the two groups. These small sample sizes (around half of what was expected) would most likely lead to underpowered study results. To contribute satisfactorily to the HTA, two more feasible trials were designed using individual randomization. This paper outlines the protocol for these trials—the Collabri Flex Trial for Depression and the Collabri Flex Trial for Anxiety. Instead of comparing collaborative care with treatment as usual, which would have been preferable, the aim is to compare collaborative care with a form of consultation liaison that contains potential contaminating elements from collaborative care (supervision and support by mental-health specialists). Because we assume that this contamination is difficult to avoid in the comparison group, we acknowledge it by adding it to the comparison group intervention. As far as we are aware, only one study has compared collaborative care with consultation liaison [27]. In that study, the population was highly selected as participants were recruited among patients with depression in the US Veterans’ Affairs Primary Care. The authors found that collaborative care was superior to consultation liaison in reducing symptoms of depression for up to 3 months, but no difference between groups was detected at 9 months.

Based on this literature, the primary hypothesis in the Collabri Flex Trial for Depression is that patients in the collaborative care group will show a greater reduction in depression symptoms after 6 months compared with patients in the consultation liaison group. The primary hypothesis in the Collabri Flex Trial for Anxiety is similar: patients in the collaborative care group will show a greater reduction in anxiety symptoms after 6 months compared with patients in the consultation liaison group.

## Methods

### Design

**Fig. 1:**
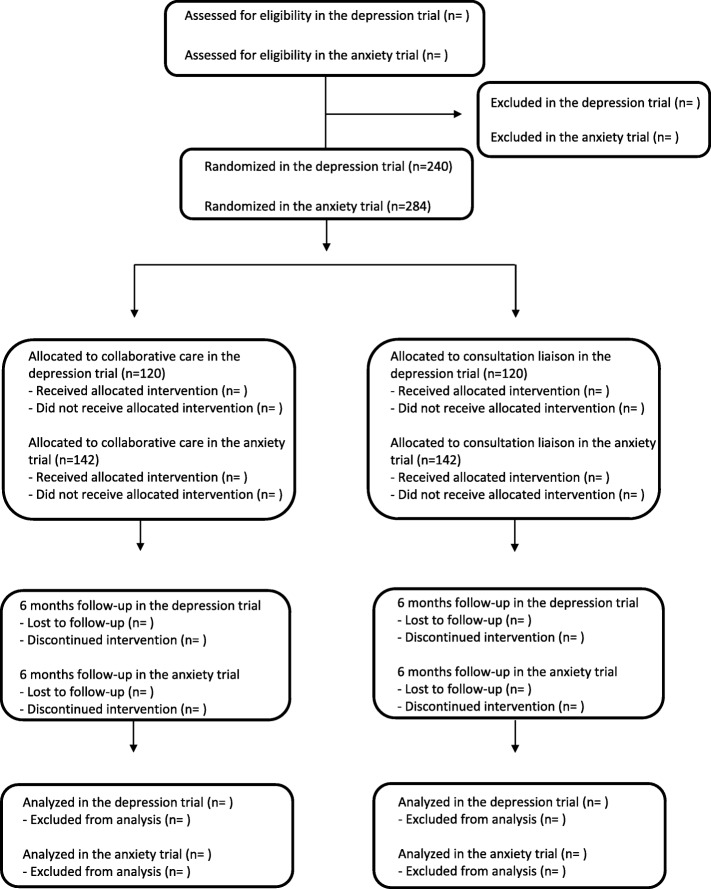
Flow chart of the trials

The Collabri Flex study involves two randomized, investigator-initiated, parallel-group superiority trials: one for the ICD-10 diagnoses of depression, and one for anxiety (the ICD-10 diagnoses of generalized anxiety, panic disorder and social anxiety disorder). The aim of the depression trial is to compare the effects of two interventions: collaborative care and consultation liaison. Similarly, the aim of the anxiety trial is to compare the effects of collaborative care with those of consultation liaison (see flow chart in Fig. 1).

### Setting

Participants are referred to one of the two trials by their GP. The GP practice must be based in the Capital Region of Denmark and the GP must agree to the terms and conditions of the study. The terms and conditions include financial reimbursement (an amount equivalent to around 80 USD per patient in the consultation liaison group and 360 USD per patient in the collaborative care group), and they are negotiated by the local branch of the Organization of General Practitioners in Denmark and the Capital Region of Denmark.

### Participants

The inclusion and exclusion criteria for both trials are assessed by the GP on recruitment and/or by the care manager at a baseline diagnostic eligibility interview. According to the inclusion criteria, eligible participants must:

- be 18 years or older
- be able to speak Danish
- give their written consent to participate
- fulfill the diagnostic criteria for unipolar depression (F32 and F33) according to the International Classification of Diseases 10th edition (ICD-10) in the depression trial, and fulfill the diagnostic criteria for social anxiety disorder, panic disorder or generalized anxiety disorder (F40.1, F41.0 and F41.1) according to the ICD-10 in the anxiety trial

All diagnoses are verified at baseline by a care manager from the Collabri Flex team using the Mini International Neuropsychiatric Interview (MINI) (Diagnostic and Statistical Manual of Mental Disorders, 4th edition) including ICD-10 specific questions, and are confirmed by the psychiatrist.

The Collabri Flex trials are aimed at patients in primary care. Consequently, the trials are not designed to provide acute or highly specialized treatment. Therefore, patients are excluded if they:

- have a high risk of suicide
- have a current psychotic condition
- have a post-traumatic stress disorder (PTSD)
- have an obsessive-compulsive disorder (OCD)
- have a bipolar affective disorder
- have a severe alcohol or substance misuse that prevents them from participating in the Collabri Flex intervention
- have been referred to or are recommended for referral to secondary care treatment (mental health center) or psychiatrist in private practice
- have been assessed by the GP as being too somatically unstable to adhere to the treatment
- are pregnant
- have a diagnosis of dementia

To prevent parallel treatment, patients are excluded if they will not allow treatment for anxiety or depression according to the psychologist scheme or similar treatment to be preceded by collaborative care treatment if they are allocated to the group offered this. Likewise, patients are excluded if they already receive treatment according to the psychologist scheme or similar treatment and indicate that they will not opt out of treatment if they are allocated to the group offered collaborative care.

### Recruitment and randomization

The GP provides oral and written information about the study, obtains oral and written consent, and refers patients with depression or anxiety to the trials. A care manager contacts the patient to arrange a diagnostic eligibility interview assessing the inclusion and exclusion criteria. If the patient meets the inclusion criteria and is not excluded, a Collabri Flex team member performs the randomization through the Odense Patient data Explorative Network (OPEN), which is an external web-based randomization provider [28]. The allocation sequence is computer generated and the block sizes are variable. The randomization in both trials is stratified by former psychological and/or pharmacological treatment for anxiety or depression (yes/no). In the depression trial, an additional stratification variable is the degree of depression as assessed in the eligibility interview (mild/moderate/severe), and in the anxiety trial an additional stratification variable is the type of anxiety disorder assessed as the primary diagnosis (generalized anxiety disorder/panic disorder/social anxiety disorder). The care manager will contact the patient with information about the result of the randomization. If the patient is allocated to collaborative care, the care manager will schedule the first consultation. If the patient is allocated to the group offered consultation liaison, the GP will continue the treatment. Ideally, the time from referral to randomization should not exceed 3 weeks (see Table 1).

**Table 1:** Standard Protocol Items: Recommendations for Interventional Trials (SPIRIT) figure: enrolment and data collection

	Baseline *t*_−1_ (0–3 weeks)	Randomization *t*_0_	6-month follow-up *t*_1_
Informed consent	x		
Eligibility interview	x		
Allocation		x	
Questionnaire data	x		x
Register data		x	x
Data on intervention activities		x	Continuous data collection throughout the intervention

### Blinding

It is not possible to ensure blinding of the allocation to patients, their GP or the Collabri Flex team, including care managers and psychiatrists involved in the intervention activities. Researchers are blinded to allocation if they contact patients at follow-up to collect data. This will be relevant only if participants require help in completing self-assessment data. During the entire phase of statistical analyses, the groups will be coded and anonymized (e.g. X and Y) so that researchers are blinded. This will also apply when writing the conclusion.

### Interventions

The Collabri Flex team consists of seven full-time care managers; they are all health-care professionals with a medium-long education and have mental health care experience and at least 1 year of certified training in CBT or equivalent. One care manager is also the team leader and ensures for example patient flow and quality improvement implementation. A psychiatrist, equivalent to a 0.9 full-time position, is also a part of the team. Additionally, an approved CBT supervisor provides 2 h of supervision every 2 weeks. Care managers have attended a 1-week introductory course to the Collabri model, which the Collabri Flex model builds upon. The course included a brush-up on CBT methods. The psychiatrist also participated in this training. All GPs are trained in the principles of collaborative care and the Collabri Flex model. Changes from the Collabri model to the Collabri Flex model have been disseminated through workshops prior to intervention start. A care manager can assist a maximum of five GP practices and hold a maximum case load of 25 patients at a time.

To ensure the internal validity and quality of the Collabri Flex intervention, an evaluation of the fidelity will be done after 6 months and at least once more during the project period. The fidelity measurement ensures that the intervention is carried out according to the description of the Collabri Flex model and will be conducted by persons who are not part of the research group. Based on the assessments, an action plan is developed to improve fidelity.

The Collabri Flex team is employed in the mental-health services and delivers two separate interventions: collaborative care according to the Collabri Flex model; and consultation liaison. The content of these interventions is outlined in the following.

#### Collaborative care

**Fig. 2:**
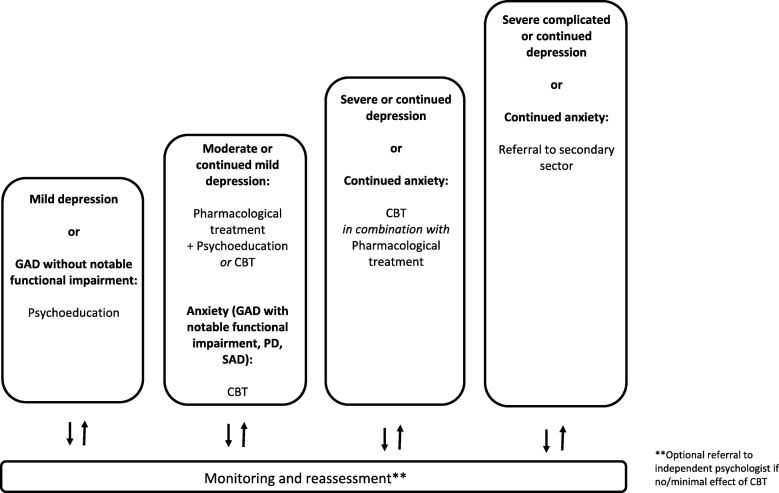
Stepped care model showing treatment modalities offered according to diagnosis and step. *CBT* cognitive behavioral therapy, *GAD* generalized anxiety disorder, *PD* panic disorder, *SAD* social anxiety disorder

The collaborative care model tested in the trials is based on the former Collabri model [24, 25] and has been updated to incorporate key knowledge and experiences from the Collabri anxiety and depression trials. The Collabri Flex model complies with the collaborative care criteria [18, 19] in the following way:

- The model proposes a multi-professional approach to treatment that involves a GP, a care manager and a psychiatrist.
- The inter-professional communication is promoted through planned, regular contact. The GP and care manager have weekly meetings. Twice a month, the psychiatrist provides supervision of care managers in planning and modifying the treatment plans. They meet individually as needed. Twice a month, care managers receive supervision from a cognitive behavioral therapy (CBT) supervisor. Once a month, GPs can participate in group-based and/or individual supervision and take part in educational workshops on specific topics. The GP, care manager and the psychiatrist can have joint consultations when needed. Preferably, communication between professionals is face to face; however, due to logistic challenges, video-conferencing is an alternative. Preferably, a joint recording system should be established, but this has not been possible in the current setting. A safe electronic communication system is used when communication containing person-identifiable information occurs between care managers/psychiatrists and the GP.
- Individual treatment plans are developed based on treatment instructions for depression, generalized anxiety disorder, panic disorder and social anxiety disorder taking into account the patient’s needs and preferences. The instructions comply with the Danish Health Authority’s Reference programs for anxiety disorders [29] and unipolar depression in adults [30] as well as the Danish College of General Practitioners’ clinical guidelines for anxiety disorders [31] and unipolar depression [32]. They include general principles of care, stepped care algorithms, medication algorithms, and psychoeducation and CBT manuals. Depending on the diagnosis and severity, patients are offered treatment modalities according to a stepped care algorithm, which offers stepwise intensification of treatment efforts if no or limited treatment response is achieved [29, 30, 32] (see Fig. 2). The treatment modalities are: manualized psychoeducation either alone or as a part of CBT, individual CBT (up to 12 sessions depending on the diagnosis) and/or medication. Additionally, all patients are offered supplementary disease-specific information material and a self-management book based on the Chronic Disease Self-Management Program (CDSMP) [33] developed for people with anxiety and depression.
- The care manager provides close scheduled follow-up of treatment progression including monitoring and reassessment to ensure timely changes in treatment plans. Monitoring occurs every 2 weeks or more frequently depending on the severity of disease. When medication is initiated, closer monitoring occurs. Reassessments take place at least once a month, at every step up and at the end of treatment.

**Fig. 3:**
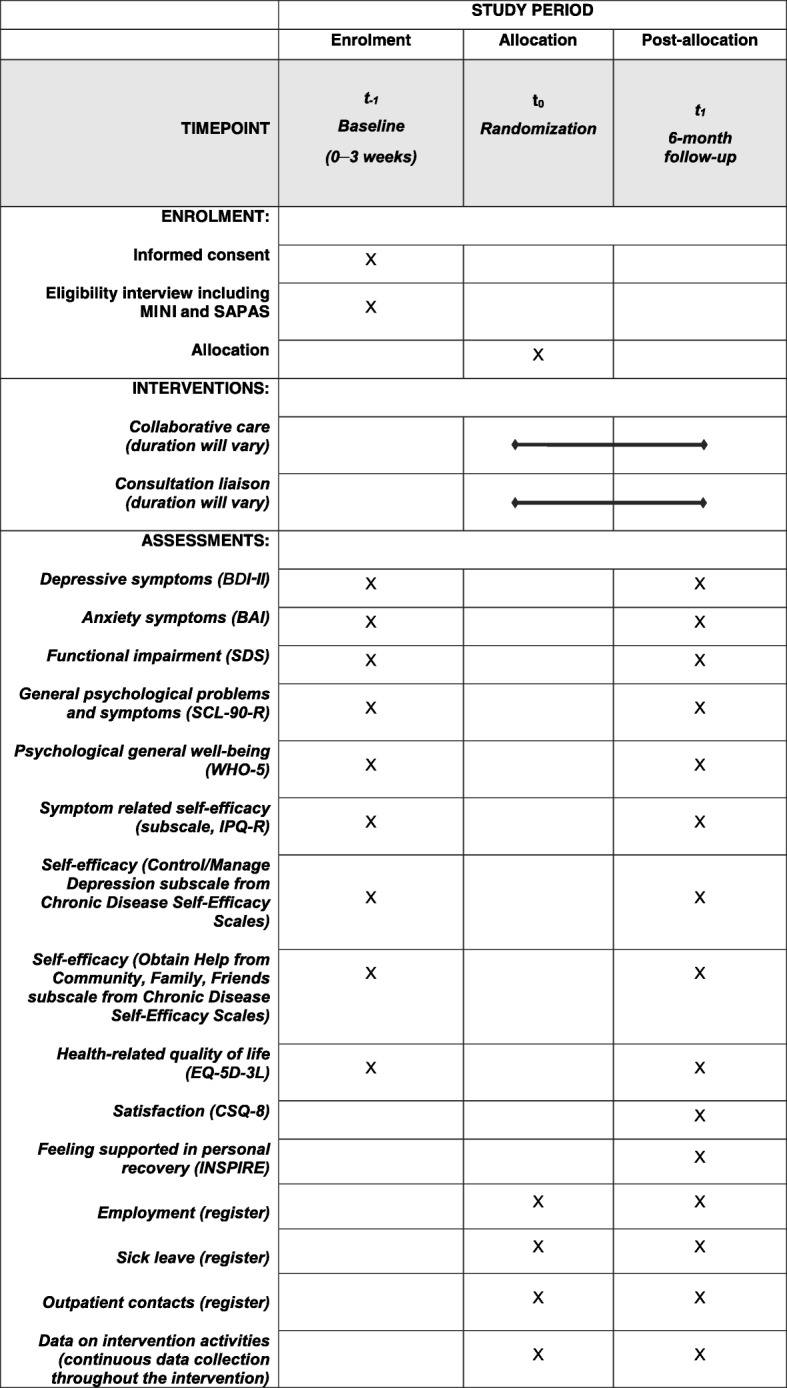
Full Standard Protocol Items: Recommendations for Interventional Trials (SPIRIT) figure. *BAI* Beck Anxiety Inventory, *BDI-II* Beck Depression Inventory II, *CSQ-8* Client Satisfaction Questionnaire with eight questions, *EQ-5D-3 L* EuroQol five-dimension three-level version of health-related quality of life, *IPQ-R* Illness Perception Questionnaire Revised, *MINI* MINI International Neuropsychiatric Interview, *SAPAS* Standardized Assessment of Personality: Abbreviated Scale, *SCL-90-R* Symptom Checklist 90-Revised, *SDS* Sheehan Disability Scale, *WHO-5* World Health Organization-Five Well-being Index

Experiences from the Collabri trials show differences between GPs in terms of interest in the treatment management. Consequently, the Collabri Flex model was developed to accommodate the needs of the GPs, thus making the GP role flexible. Apart from the somatic evaluation and pharmacological treatment, the GP can pass on the treatment responsibility to the psychiatrist. If this is the case, it is agreed under which circumstances the GP is involved. As a minimum, the GP is informed about and approves the treatment plan and potential changes.

The model is adapted to the Danish health-care setting by incorporating the possibility of referral to the existing psychologist scheme if there is no or minimal effect of CBT and the possibility of coordination with relevant social workers in the municipalities. Relatives/friends are offered disease-specific written information material including relevant links and contact details for further information and advice. It is also possible for a relative/friend to attend a care manager consultation. The model incorporates patient involvement activities, especially with the aim of facilitating shared decision-making, and strives to engage patients in self-management of their disease.

It is the intention that patient consultations take place in the GP’s clinic; however, due to logistic challenges, not all GPs have the capacity. In these cases, care managers can meet patients in community health centers, mental-health centers or other places in agreement with the patient and the GP.

The estimated average duration of the collaborative care intervention is 4 months, depending on the severity of disease and any need to intensify treatment (step up). There is no predefined minimum or maximum duration of the intervention.

#### Consultation liaison

Consultation liaison performed in the present trials falls under a narrow definition as suggested by Cape et al. [16]: the Collabri Flex team can provide guidance to the GP but does not have any treatment-related contact with patients after the diagnostic assessment. All GPs are encouraged to participate in the monthly supervision and educational activities facilitated by the psychiatrist. In addition to the scheduled meetings, the GPs can consult the Collabri Flex team about patient-specific concerns regardless of the patient’s allocation. For example, this could involve diagnostic-related, treatment-related or referral-related guidance and support. For patients allocated to consultation liaison, their GP maintains the treatment responsibility and continues to manage their care. GPs can use disease-specific guidelines from the Danish Health Authority and the Danish College of General Practitioners [29–32]. If relevant, GPs can prescribe medication and provide a limited number of therapy sessions. However, they can only provide therapy if they attend supervision [34]. It is estimated that only around one-third of GPs in Denmark can provide therapy [12, 35, 36]. GPs can refer patients to an independent psychologist (partly publicly subsidized), an independent psychiatrist (fully publicly subsidized) or mental-health services (fully publicly subsidized). Patients with mild to moderate anxiety (including generalized anxiety, panic disorder or social anxiety disorder) and aged between 18 and 38 years and all patients with mild to moderate depression can attend up to 24 sessions of partly publicly subsidized therapy if they see an independent psychologist [34]. Mental-health services offer outpatient treatment including diagnostic assessment, pharmacological- and non-pharmacological treatment involving up to 14 sessions of therapy in groups or seven individual sessions [37–39]. Similarly, outpatient treatment is part of the Collabri Flex model because it constitutes the last step in the stepped care models (see Fig. 2). Accessible treatment can vary between general practices as guidelines provide only recommendations for treatment; therefore, the estimated duration of treatment can vary accordingly.

### Data collection and data management

Data will consist of information obtained by the Collabri Flex team (from baseline throughout the study period), self-reported questionnaire data and register data.

The baseline diagnostic eligibility interview is supervised by and delegated by the psychiatrist. The diagnostic assessment is based on the Mini International Neuropsychiatric Interview (MINI) [40], in which all care managers have received extensive training. The psychiatrist is always consulted to discuss the assessment and clinical impression. If there is disagreement between the result of the diagnostic assessment and the GP’s referral diagnosis, the psychiatrist contacts the GP to agree on the primary diagnosis and potential secondary diagnoses. In connection with the diagnostic assessment, the care manager obtains additional baseline information using the Standardized Assessment of Personality Abbreviated Scale (SAPAS) [41] and, if relevant, the Attention-Deficit/Hyperactivity Disorder Symptom Checklist for Adults (Adult ADHD Self-report Scale (ASRS)) [42]. Self-reported questionnaires will be completed electronically before the diagnostic assessment and 6 months after randomization.

Diagnostic assessment data, intervention-specific data and self-reported questionnaire data are collected through and stored in the electronic system REDCap [43], which is the data management system required by the Capital Region of Denmark. Other data, such as data extractions for quality assurance, are stored in entry-restricted files on secured and logged servers. Blinding will be maintained.

### Outcomes

In the depression trial, the primary outcome is symptoms measured by the Beck Depression Inventory (BDI-II) [44]. The BDI-II is a self-reported 21-item general depression questionnaire, measuring depression symptoms during the past 14 days. Symptoms are rated on a 4-point Likert scale ranging from 0 to 3. In a review of the psychometric properties of the BDI-II, the authors reported a high internal consistency (Cronbach’s α ranging from 0.83 to 0.96) and a test–retest reliability (Pearson’s *r*) ranging from 0.73 to 0.96 [45]. In primary care patients, the questionnaire has shown reliable and internally consistent scores [46].

In the anxiety trial, the primary outcome is symptoms measured by the Beck Anxiety Inventory (BAI) [47]. The BAI is a self-reported 21-item general anxiety questionnaire, measuring anxiety symptoms during the past week. Symptoms are rated on a 4-point Likert scale ranging from 0 (never) to 3 (almost all the time). The questionnaire has demonstrated high internal consistency (Cronbach’s α = 0.92) and a 1-week test–retest reliability of 0.75 in a group of outpatients mainly with anxiety or depression [48]. In a primary care setting, the BAI has been shown to reflect the severity of anxiety in patients with different anxiety disorders [49].

In both trials, the self-reported secondary outcomes are general psychological problems and symptoms measured by the Symptom Checklist (SCL-90-R) [50], functional impairment measured by the Sheehan Disability Scale (SDS) [51–53] and well-being measured by the World Health Organization-Five Well-Being Index (WHO-5) [54, 55]. In the anxiety trial, the BDI-II is an additional secondary outcome, and the BAI is an additional secondary outcome in the depression trial.

The SCL-90-R is a questionnaire designed to assess a broad range of general psychological problems and symptoms. This multi-dimensional questionnaire consists of 90 items rated on a 5-point Likert scale ranging from 0 (not at all) to 4 (extremely). The items are divided into nine subscales (depression, anxiety, phobic anxiety, obsession/compulsion, hostility, somatization, interpersonal sensitivity, paranoid ideation and psychoticism) from which the joint measure, the Global Severity Index (GSI), can be calculated as the average score of the 90 items.

The SDS includes three items and measures functional disability in relation to work, social and family life. For each item there are 11 potential responses reflecting the degree of impairment, ranging from 0 (not at all) to 10 (extremely). A total score ranging from 0 (not impaired) to 30 (highly impaired) can be calculated.

The WHO-5 includes five items measuring the experience of positive psychological well-being. Each item is rated from 0 (not present) to 5 (constantly present) on a 6-point Likert scale.

Explorative outcomes in both trials are self-reported health-related quality of life measured by the EuroQol Five Dimensions Questionnaire (EQ-5D-3 L) [56]; self-efficacy measured by the *Personal Control* subscale from the Illness Perception Questionnaire Revised (IPQ-R) [57] and two subscales (*Obtain Help from Community, Family, Friends Scale* and *Control/Manage Depression Scale*) from the Chronic Disease Self-Efficacy Scales [58]; experience of support in personal recovery measured by INSPIRE [59]; general satisfaction with treatment measured by the Client Satisfaction Questionnaire (CSQ-8) [60, 61]; sick leave; employment; and outpatient mental-health services obtained from registers (see Table 2 and Fig. 3 for an overview of primary, secondary and explorative outcomes). The EQ-5D-3 L measures health-related quality of life in five domains: mobility, self-care, usual activities, pain/discomfort and anxiety/depression. The assessment also contains a visual analog scale ranging from 0 (worst imaginable health status) to 100 (best imaginable health status). The IPQ-R consists of 12 subscales and the *Personal Control* subscale is a six-item scale reflecting a person’s beliefs about their ability to affect own symptoms. The items are rated on a 5-point Likert scale ranging from 1 (disagree very much) to 5 (agree very much). The *Obtain Help from Community, Family, Friends* subscale from the Chronic Disease Self-Efficacy Scales consists of four items about how confident the person feels in getting emotional support and help with daily tasks from the community, family and friends. The *Control/Manage Depression* subscale from the Chronic Disease Self-Efficacy Scales consists of six items regarding how confident the person feels about doing something to feel better when feeling sad, discouraged or lonely. Each item on these two subscales is rated on a 10-point Likert scale ranging from 1 (not at all confident) to 10 (very confident). The INSPIRE questionnaire measures the patients’ feelings of being supported in their recovery by their primary health-care provider(s). The questionnaire has two sections: one assessing support from the health-care provider (20 items); and one assessing the relationship with the health-care provider (seven items). General satisfaction with treatment is assessed through the CSQ-8 questionnaire, which consists of eight items rated on a scale ranging from 1 to 4. Information about sick leave and employment is obtained from the Danish Register for Evaluation of Marginalization (DREAM) database [62]. Information about outpatient mental-health contacts is collected through the National Patient Register, which contains information about all patient contacts in the secondary health-care system [63].

**Table 2:** Outcomes, data source and time for data collection

Data source	Outcome		Baseline	6-month follow-up
Questionnaire	Anxiety symptoms measured by the Beck Anxiety Inventory (BAI)	Primary outcome in the anxiety trial, secondary outcome in the depression trial	x	x
Questionnaire	Depression symptoms measured by the Beck Depression Inventory (BDI-II)	Primary outcome in the depression trial, secondary outcome in the anxiety trial	x	x
Questionnaire	Functional impairment measured by the Sheehan Disability Scale (SDS)	Secondary outcome	x	x
Questionnaire	General psychological problems and symptoms measured by the Symptom Checklist (SCL-90-R)	Secondary outcome	x	x
Questionnaire	Psychological general well-being measured by the World Health Organization-Five Well-being Index (WHO-5)	Secondary outcome	x	x
Questionnaire	Self-efficacy related to symptoms measured by the *Personal Control* subscale from the Illness Perception Questionnaire Revised (IPQ-R)	Explorative outcome	x	x
Questionnaire	Self-efficacy related to management of disease measured by the *Control/Manage Depression* subscale from the Chronic Disease Self-Efficacy Scales	Explorative outcome	x	x
Questionnaire	Self-efficacy related to support from others measured by the *Obtain Help from Community, Family, Friends* subscale from the Chronic Disease Self-Efficacy Scales	Explorative outcome	x	x
Questionnaire	Health-related quality of life measured by the EuroQol five-dimension three-level version of health-related quality of life (EQ-5D-3L)	Explorative outcome	x	x
Questionnaire	Satisfaction with treatment measured by the Client Satisfaction Questionnaire (CSQ-8)	Explorative outcome		x
Questionnaire	Feeling of being supported in personal recovery by the primary health-care provider measured by the INSPIRE measure	Explorative outcome		x
DREAM database	Sick-leave benefit (yes/no) at follow-up and weeks on sick-leave benefit from baseline to follow-up	Explorative outcomes	x	x
DREAM database	Employment (yes/no) at follow-up and weeks in employment from baseline to follow-up	Explorative outcomes	x	x
National Patient Register	Mental-health outpatient contacts from baseline to follow-up	Explorative outcome	x	x

*DREAM* Danish Register for Evaluation of Marginalization

### Other data collection

Descriptive information about sex, age, housing and education is collected through Statistics Denmark, which is the central authority on Danish statistics [64]. Information about workforce participation is collected through the DREAM database [62]. Information about former treatment for anxiety or depression is collected through the National Patient Register [63] and information about screening for personality disorder is based on the Standardized Assessment of Personality (SAPAS) [41].

Collaborative care-specific data, such as use of treatment modalities, number of step-ups and sessions, will be registered by the Collabri Flex team throughout the intervention period. Information about consultations between the patient and GP will be registered in both the collaborative care and consultation liaison group at 1, 3 and 6 months and patient-specific communication between professionals will be registered continuously.

### Safety measures

Safety measures are self-reported anxiety and depression symptoms measured by the BAI [47] and BDI-II [44]; deaths from suicide and other causes collected through the Danish Register of Causes of Death [65]; and somatic inpatient and outpatient services and psychiatric inpatient services obtained from the National Patient Register [63].

### Sample size and power calculations

#### The Collabri Flex Trial for Depression

Clinically relevant treatment response at group level is defined as a 4-point difference in depression symptoms measured by the BDI-II [66, 67]. At the time of sample size calculation, we found no relevant Danish studies that could contribute to the estimation of the within-group standard deviation (SD) for the BDI-II in this population. Therefore, the SD for the BDI-II is set to 11, based on international surveys [66–68]. A sample size calculation based on these figures shows that 240 participants should be included to be able to reject the null hypothesis that the collaborative care group and the consultation liaison group have improved similarly in terms of symptoms with a power of 0.8 and a significance level of 0.05.

#### The Collabri Flex Trial for Anxiety

Clinically relevant treatment response is defined as a 4-point difference in anxiety symptoms measured with the BAI. The estimation is based on studies using the BDI [66, 67], because, to our knowledge, there are no comparable Danish studies using the BAI. Additionally, we could not find any Danish studies that could contribute to the estimation of the SD for the BAI in this population. Therefore, the SD is set to 12 based on international surveys [68–70]. Based on these figures, the sample size calculation shows that 284 individuals should be included in the anxiety trial to be able to reject the null hypothesis that the collaborative care group and the consultation liaison group have improved similarly in terms of anxiety symptoms with a power of 0.8 and a significance level of 0.05.

Power calculations for the secondary outcomes showed that 120 participants in each group in the depression trial and 142 participants in each group in the anxiety trial will be sufficient to detect relevant significant differences in the secondary outcomes with a power above 80% (see Tables 3 and 4). The power calculations were performed subsequent to the sample size calculations. Some of the figures are based on Collabri data that were not available at the time of sample size calculations.

**Table 3:** Power calculations for secondary outcomes in the depression trial

	δ value for clinically relevant difference	α value for type I error	σ value for the within-group SD	Calculated power	Reference
BAI	4	0.05 (5%)	10	0.870 (87.0%)	[66, 67] SD is based on data from the Collabri depression trial (not published)
SDS	4	0.05 (5%)	9	0.930 (93.0%)	[51, 52, 71]
SCL-90-R	23	0.05 (5%)	50	0.944 (94.4%)	[72]
WHO-5	10	0.05 (5%)	18	0.990 (99.0%)	[55, 73] SD is based on data from the Collabri depression trial (not published)

*BAI* Beck Anxiety Inventory, *SCL-90-R* Symptom Checklist 90-Revised, *SD* standard deviation, *SDS* Sheehan Disability Scale, *WHO-5* World Health Organization-Five Well-being Index

**Table 4:** Power calculation for secondary outcomes in the anxiety trial

	δ value for clinically relevant difference	α value for type I error	σ value for the within-group SD	Calculated power	Reference
BDI-II	4	0.05 (5%)	11	0.863 (86.3%)	[66–69]
SDS	4	0.05 (5%)	10	0.920 (92.0%)	[51, 52, 71]
SCL-90-R	23	0.05 (5%)	50	0.972 (97.2%)	[72]
WHO-5	10	0.05 (5%)	26	0.898 (89.8%)	[55, 73] SD is based on data from the Collabri anxiety trial (not published)

*BDI-II* Beck Depression Inventory II, *SCL-90-R* Symptom Checklist 90-Revised, *SD* standard deviation, *SDS* Sheehan Disability Scale, *WHO-5* World Health Organization-Five Well-being Index

### Statistical analyses

The null hypothesis tested in both trials is that there is no difference in depression and anxiety symptoms, respectively, between the two groups (collaborative care and consultation liaison) at 6 months’ follow-up. For continuous outcomes, including the primary outcomes, analysis of variance (ANCOVA) will be used to examine differences between group means. For exploratory binary outcomes, logistic regression analysis will be used to detect differences between groups. All analyses will be adjusted for stratification variables. The trials will be conducted according to the statistical principle “intention-to-treat”, which means that analyses will be based on all included participants. If necessary, multiple imputations will be used to address the issue of missing data.

## Discussion

The design of the trials has several strengths. The collaborative care model has been developed based on previous experiences and with special emphasis on meeting the needs of GPs. We hope this will increase the success of any subsequent implementation; fidelity reviews will be conducted to ensure faithfulness to the intervention; we recognize the risk of contamination of the control group when testing a system-level intervention, and therefore we compare the collaborative care group with a form of consultation liaison that equals the contaminating elements; the randomization is externally computer generated, which reduces the risk of selection bias; compared with the Collabri trials, the questionnaire battery has been shortened to enhance the follow-up rate; and the psychiatrist in the Collabri Flex team and many of the care managers and GPs also participated in the previous Collabri trials, and, accordingly, they have already worked within the framework of the similar Collabri model and have experienced being part of an RCT.

The design of the trials also has some limitations. Most outcomes, including the primary outcomes, are self-reported, which could lead to information bias and, possibly, overestimation of effects [74]; participants, the Collabri Flex team and GPs are not blinded to the allocation, which could lead to performance bias; because we compare two active intervention groups, it might be more difficult to detect a difference between groups than if we had compared collaborative care with treatment as usual; thus, accordingly, from these trials we will not be able to conclude whether collaborative care is more effective than treatment as usual. However, the results will supplement those of the cluster-randomized Collabri trials and together they will provide pivotal information about the effects of collaborative care in Denmark.

## Trial status

The Collabri Flex Trial for Depression and the Collabri Flex Trial for Anxiety were initiated in mid-January 2018. Recruitment is ongoing and expected to end 12 months after trial initiation. The results are expected after an additional 12 months. This protocol is version 2.

## Additional file

Additional file 1: SPIRIT 2013 Checklist: Recommended items to address in a clinical trial protocol and related documents (DOC 124 kb)

## Data Availability

Not applicable.

## Abbreviations

ASRS: Adult ADHD Self-report Scale
BAI: Beck Anxiety Inventory
BDI-II: Beck Depression Inventory II
CBT: Cognitive behavioral therapy
CDSMP: Chronic Disease Self-Management Program
CSQ-8: Client Satisfaction Questionnaire with eight questions
DREAM: Danish Register for Evaluation of Marginalization
EQ-5D-3 L: EuroQol five-dimension three-level version of health-related quality of life
GP: General practitioner
IPQ-R: Illness Perception Questionnaire Revised
MINI: Mini International Neuropsychiatric Interview
OPEN: Odense Patient data Explorative Network
SAPAS: Standardized Assessment of Personality: Abbreviated Scale
SCL-90-R: Symptom Checklist 90-Revised
SDS: Sheehan Disability Scale
WHO-5: World Health Organization-Five Well-being Index
